# The Cellular Effects of Di(2-ethylhexyl) Phthalate in Non-Malignant Colonic Epithelia Involve Oxidative Stress

**DOI:** 10.3390/ijms262311716

**Published:** 2025-12-03

**Authors:** Zachary S. Bomstein, Kimberly F. Allred, Clinton D. Allred

**Affiliations:** Department of Nutrition, University of North Carolina at Greensboro, 319 College Ave., Greensboro, NC 27412, USA; zsbomstein@uncg.edu (Z.S.B.); kfallred@uncg.edu (K.F.A.)

**Keywords:** Di(2-ethylhexyl) phthalate (DEHP), aryl hydrocarbon receptor (AhR), oxidative stress, nuclear factor erythroid 2-related factor 2 (Nrf2), colonocytes

## Abstract

Human exposure to Di(2-ethylhexyl) Phthalate (DEHP) occurs through ingestion of contaminated food. Yet, the effects of DEHP on gastrointestinal toxicity at the cellular level are poorly understood and studies conducted to date have used malignant cell lines, limiting our understanding of molecular signaling in intestinal epithelia of otherwise healthy individuals. The objective of our study was to use a non-transformed, colonic epithelial cell line (Young Adult Mouse Colonocytes; YAMCs) to characterize the in vitro effects of DEHP on non-malignant colonic epithelia. A 72 h DEHP exposure significantly reduced cell number and proliferation while short-term exposure increased: cellular apoptosis, *BAX* expression, Reactive Oxygen Species (ROS) production, gene expression linked to oxidative stress (*NRF2*, *GCLC*, *HO-1*, *CHOP*). Antioxidant pretreatment prior to DEHP exposure attenuated the phthalate’s apoptotic effect, suggesting a link between oxidative stress and apoptosis. Using YAMCs with a CRISPR-deleted Aryl Hydrocarbon Receptor (AhR) we further showed that the apoptotic and pro-oxidative effects of the phthalate are partially mediated through AhR. In conclusion, we have demonstrated that DEHP-induced toxicity in non-malignant colonocytes is due to ROS-induced oxidative stress and subsequently, apoptosis. We have further demonstrated that these effects are partly mediated by the AhR, a mechanism that deserves further investigation. Future studies should build on these findings by (a) characterizing the specific mechanisms linking ROS production to apoptosis demonstrated in our model of exposure, (b) measuring the dynamics of the receptor following DEHP exposure and (c) examining these effects over a longer exposure period.

## 1. Introduction

Phthalates are a diverse group of compounds used in adhesives, lubricants, fragrances, oils, and plastic polymers [1]. One such phthalate, Di(2-Ethylhexyl) Phthalate (DEHP), is used principally in the production of Polyvinyl Chloride-based plastics, like those utilized in medical tubing/devices, food packaging, beverage bottles, upholstery, carpeting, and vinyl flooring [2]. The International Agency for Research on Cancer (IARC), the U.S. Environmental Protection Agency (EPA), and The U.S. Department of Health and Human Services (DHHS) all deem DEHP to be a possible human carcinogen and concerns over its endocrine-disrupting activity and reproductive toxicity have resulted in regulatory agencies like the U.S. Consumer Product Safety Commission (CPSC) and the European Chemicals Agency (ECHA) limiting its usage in plastics that come into contact with humans [3,4]. Despite these regulations, human exposure to DEHP persists due to (a) its broad usage in food packaging/processing materials, (b) variability regarding its regulations in countries worldwide, and (c) its environmental longevity, allowing for its survival in water, soil, and other matrices for extensive periods of time [5,6,7,8]. DEHP’s usage in plastic food packaging/processing materials, coupled with its weak linkage to plastic polymers means that the phthalate is easily shed into the external environment. Consequently, humans are exposed to DEHP principally through the ingestion of contaminated food and as such, the organs of the gastrointestinal tract are primary targets of DEHP toxicological effects [9,10]. However, the majority of studies investigating DEHP’s toxicity have focused on its biological effects on tissues such as those involved in reproductive, neurological, hepatic, cardiovascular, and metabolic health while the effect of DEHP on organs of the gastrointestinal tract is poorly understood [3].

The large intestine is the final organ of the gastrointestinal (GI) tract and due to this positioning, is exposed to orally consumed toxicants as well as bioactive compounds excreted by the host, including phthalates such as DEHP and its metabolites [11,12]. The layer of epithelial cells, mucus, and anchored microorganisms lining its interior play critical roles in regulating the influx of nutrients, and fluids, and maintenance of the protective intestinal barrier meant to preserve its integrity [11,13]. Consequently, studying molecular actions of compounds on cells derived from the colonic epithelium provides critical insights into how their effects might translate to tissue-wide responses [14].

To this end, several in vitro studies have sought to characterize the effect of DEHP in culture systems derived from colonic tissue with conflicting results. In HCT-116 cells, DEHP dose-dependently reduced cellular viability, damaged mitochondrial function, increased oxidative stress, and induced apoptosis [15]. Conversely, SW620 cells pretreated with DEHP for two months exhibited greater resistance to 5-fluorouracil-mediated apoptosis, enhancing cell survival through manipulating oxidative phosphorylation [16]. It is important to note that both of these cell lines are malignantly transformed. That is important because differences in mutations to key oncogenic or tumor suppressor genes could contribute to the variability of these findings [15,16,17]. Furthermore, these results likely do not represent the cellular impact that DEHP might have on the colon in healthy individuals. Thus, understanding the effects of DEHP exposure in non-transformed, non-malignant colonocytes is critical to understanding the contribution of the phthalate to biological processes that precede the development of diseases that affect the colon. To our knowledge, no studies to date have investigated the in vitro effect of DEHP on the colon in non-cancerous, non-transformed cells. The objective of our study was to determine the in vitro effects of DEHP on non-malignant colonocytes, and to investigate mechanisms by which the phthalate might affect biological processes and signaling implicated in gastrointestinal disease.

## 2. Results

### 2.1. DEHP Exposure Reduces Cell Number and Cellular Proliferation in Non-Malignant Colonocytes

**Figure 1:**
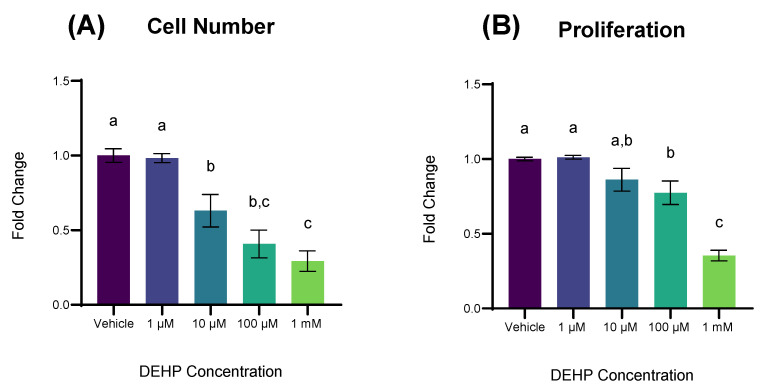
The effect of DEHP on cell number and proliferation. (**A**) YAMCs were exposed to increasing concentrations of DEHP for 72 h and cells across 25 representative areas of each well were microscopically counted using a Cytation 5 Imaging Machine (Agilent Biotek; Winooski, VT, USA). (**B**) YAMCs were exposed to increasing concentrations of DEHP for 72 h and BrdU incorporation was colorimetrically quantified with a spectrophotometer (Agilent Biotek; Winooski, VT, USA). Data are expressed as fold change relative to vehicle (0.1% *v*/*v* DMSO). Mean (n = 9) biological replicates ± SEM from three replicate experiments. Bars without a common letter are significantly different; *p* < 0.05.

First, we measured the effect of varying concentrations of DEHP on cell growth (Figure 1A). Concentrations of 10 µM, 100 µM, and 1 mM DEHP resulted in a statistically significant decrease in cell number after 72 h compared to vehicle control (*p* = 0.0074, *p* < 0.000 and *p* < 0.0001, respectively). Next, we measured proliferation in cells exposed to varying concentrations of DEHP using a Bromodeoxyuridine (BrdU) Proliferation kit (Millipore Sigma; Burlington, MA, USA). A 72 h exposure to concentrations of 100 µM, and 1 mM DEHP resulted in a statistically significant decrease in cellular proliferation compared to vehicle control (*p* = 0.0059 and *p* < 0.0001, respectively; Figure 1B).

### 2.2. Short-Term Exposure to DEHP Results in Apoptosis but Not Cytotoxicity

**Figure 2:**
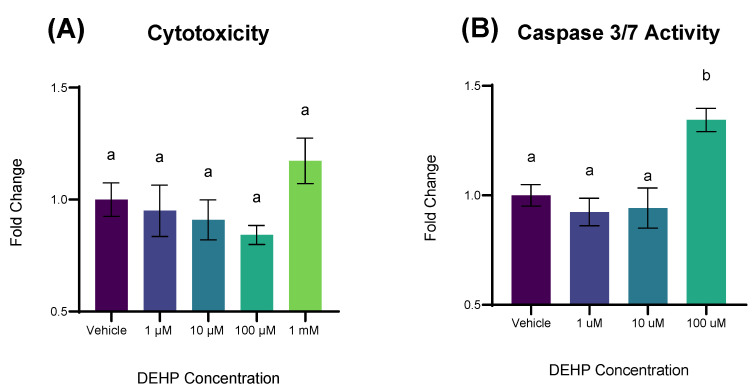
Cytotoxicity and apoptosis in YAMCs treated with DEHP. (**A**) YAMCs were exposed to varying concentrations of DEHP for 24 h. Lactate dehydrogenase in culture media was colorimetrically quantified using a spectrophotometer (Agilent Biotek; Winooski, VT, USA). (**B**) YAMCs were exposed to varying concentrations of DEHP for 12 h and caspase activity was measured on a luminometer (Agilent Biotek; Winooski, VT, USA). Data are expressed as fold change relative to vehicle (0.1% *v*/*v* DMSO). Mean (n = 9) biological replicates ± SEM from three replicate experiments. Bars without a common letter are significantly different; *p* < 0.05.

We then sought to determine whether the growth inhibitory and antiproliferative effects of DEHP over 72 h were a product of either apoptosis or cytotoxicity in the short-term. A 24 h exposure of 1 µM, 10 µM, 100 µM, and 1 mM DEHP did not result in statistically significant differences in cytotoxicity compared to vehicle control (Figure 2A). On the other hand, a 12 h exposure to 100 µM DEHP resulted in a significant increase in cleaved caspase 3/7 activity relative to vehicle control (*p* = 0.0045; Figure 2B).

### 2.3. DEHP Exposure Differentially Affects the Expression of Genes Associated with Apoptosis and Cell Survival

**Figure 3:**
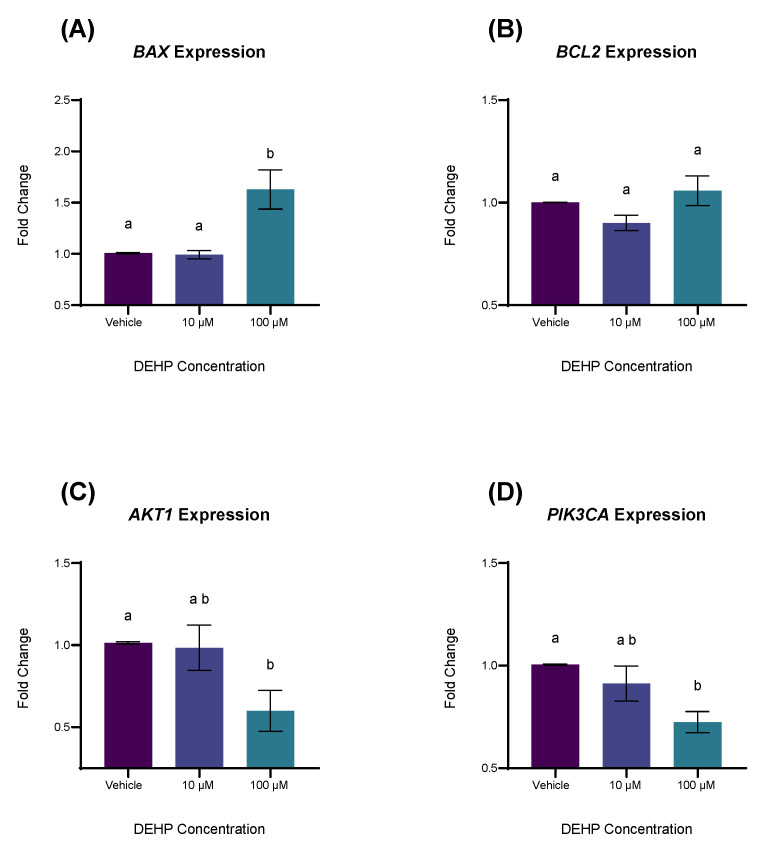
The effect of DEHP on the expression of genes involved with apoptosis (**A**,**B**) and cell survival (**C**,**D**). YAMCs were treated with varying concentrations of DEHP for 24 h. Amplification was measured using SYBR green reagents. Gene expression was normalized by 18S. Values are mean (n = 3) biological replicates ± SEM from three replicate experiments. Bars without a common letter are significantly different; *p* < 0.05.

To better understand the mechanisms underpinning the growth inhibitory and apoptotic effects of DEHP we used RT-qPCR to measure the expression of genes involved with apoptosis and signal transduction pathways important in cellular growth and survival. A 24 h exposure at concentrations of 100 µM DEHP increased the expression of pro-apoptotic gene BCL2-associated X protein (*BAX*) (*p* = 0.0188) but did not significantly alter the expression of anti-apoptotic gene B-cell lymphoma-2 (*BCL2*) (*p* = 0.6923) compared to vehicle control (Figure 3A,B). At concentrations of 100 µM DEHP significantly reduced the expression of the gene coding for the 85 kDa regulatory subunit of the PI3K protein, phosphatidylinositol-4,5-bisphosphate 3-kinase catalytic subunit alpha (*PIK3CA*) (*p* = 0.0321) and led to a borderline significant decrease in expression of the gene thymoma viral proto-oncogene 1 (*AKT1*) (*p* = 0.0971), coding for the proliferative AKT protein (Figure 3C,D).

### 2.4. DEHP Exposure Increases the Expression of Genes Involved in Antioxidant Defense and Oxidative Stress

**Figure 4:**
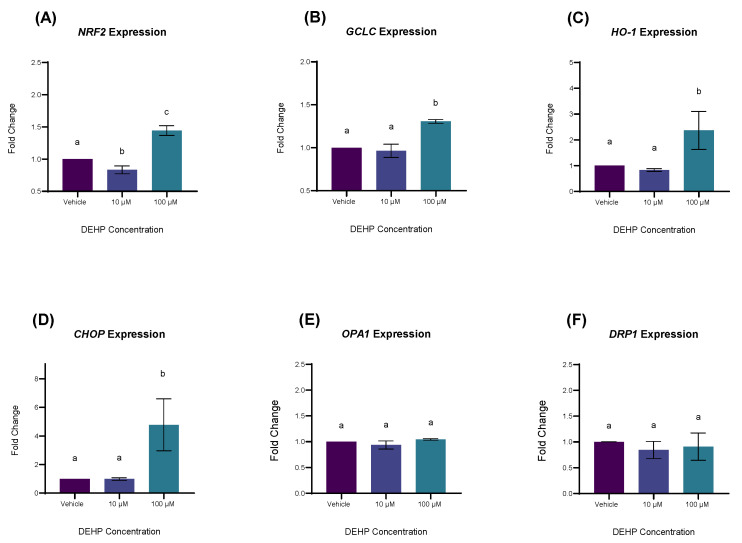
The effect of Dehp on the expression of genes involved with oxidative stress (**A**–**C**), endoplasmic reticulum stress (**D**), and mitochondrial fission (**E**,**F**). YAMCs were treated with varying concentrations of DEHP for 24 h. Amplification was measured using SYBR green reagents. Gene expression was normalized by 18S. Values are mean (n = 3) biological replicates ± SEM from three replicate experiments. Bars without a common letter are significantly different; *p* < 0.05.

The introduction of xenobiotics to the cell can promote oxidative metabolite production, which over time, may result in organelle/protein damage and eventual apoptosis [18]. In response to such disturbances, the oxidant-sensitive cytoplasmic protein nuclear factor erythroid 2-related factor 2 (Nrf2) translocates to the nucleus, forming a heterodimeric complex with small Maf proteins (sMafs) which binds to Antioxidant Response Elements (AREs), promoting the transcription of myriad genes involved in mounting a response to oxidative stress [19]. Therefore, we used Quantitative reverse transcription polymerase chain reaction (RT-qPCR) to measure the expression of Nrf2 target genes glutamate-cysteine ligase, catalytic subunit (*GCLC*), and heme oxygenase 1 (*HO-1*), as well as the expression of the *NRF2* gene itself. Further, we measured the expression of genes indicative of endoplasmic reticulum stress (DNA-damage inducible transcript 3; *CHOP*) and mitochondrial permeability (dynamin-related protein 1; *DRP1* and optic atrophy 1; *OPA1*), significant of ROS-induced organelle damage, a consequence of uncontrolled oxidative stress [20,21]. At concentrations of 100 µM DEHP significantly increased the expression of the *NRF2* and *GCLC* (*p* = 0.0033 and *p* = 0.0254, respectively; Figure 4A,B). Further, 100 µM DEHP significantly increased the expression of *HO-1*, though this difference was significant relative to 10 µM DEHP only (*p* = 0.0438; Figure 4C). At a concentration of 100 µM DEHP significantly increased the expression of *CHOP* (*p* = 0.0439) but did not significantly affect the expression of *OPA1* or *DRP1* (*p* = 0.7847 and *p* = 0.9298, respectively; Figure 4D–F).

### 2.5. DEHP-Induced Apoptosis Is Accompanied by ROS Production and Is Attenuated by NAC Pretreatment

**Figure 5:**
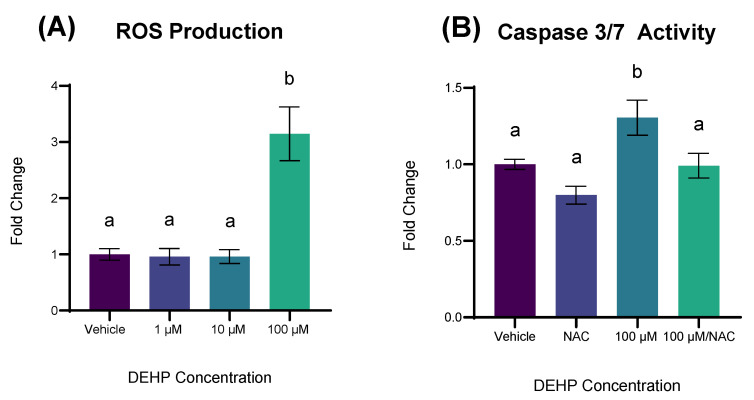
ROS production and NAC-attenuation of caspase activity after DEHP exposure. (**A**) YAMCs were exposed to varying concentrations of DEHP for 24 h. ROS activity was measured using the fluorescent probe DCFH-DA on a fluorescence plate reader (Agilent Biotek; Winooski, VT, USA). (**B**) YAMCs were exposed to varying concentrations of DEHP for 12 h, with or without N-Acetyl Cysteine (NAC) pretreatment. Caspase activity was measured on a luminometer (Agilent Biotek; Winooski, VT, USA). Data are expressed as fold change relative to vehicle (0.1% *v*/*v* DMSO). Mean (n = 9) biological replicates ± SEM from three replicate experiments. Bars without a common letter are significantly different; *p* < 0.05.

Having shown that the pro-apoptotic effects of DEHP exposure towards colonocytes were accompanied by transcriptional indices of oxidative stress, we next determined whether short-term DEHP exposure resulted in the formation of generalized Reactive Oxygen Species (ROS). A 24 h exposure to DEHP at concentrations of 100 µM increased the production of ROS in non-malignant colonocytes, compared to vehicle control (*p* < 0.0001, Figure 5A). In light of this finding, we then sought to determine whether the apoptosis in YAMCs following DEHP exposure was a product of ROS production. Cells were pre-treated with the ROS-scavenging compound N-Acetyl Cysteine (NAC) for 2 h prior to DEHP exposure and caspase activity was measured as an approximation of apoptosis. NAC pretreatment to YAMCs exposed to 100 µM DEHP significantly reduced caspase activity relative to cells exposed to 100 µM DEHP alone (*p* = 0.0307; Figure 5B)

### 2.6. Knock-Out of the Aryl Hydrocarbon Receptor Disrupts Apoptotic Signaling in YAMCs Exposed to DEHP

**Figure 6:**
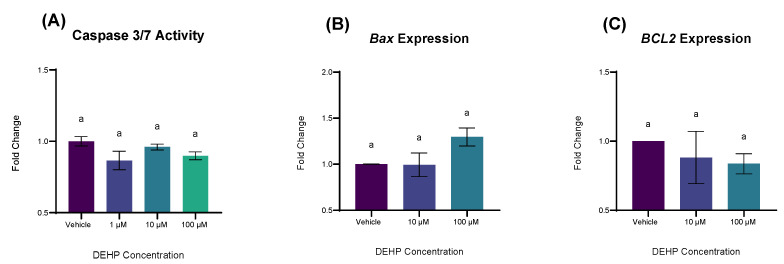
The effect of DEHP on Caspase 3/7 activity and transcriptional measures of apoptosis in YAMC-AhR-KOs. (**A**) YAMC-AhR-KOs were exposed to varying concentrations of DEHP for 12 h and caspase activity was measured on a luminometer (Agilent Biotek; Winooski, VT, USA). Mean (n = 9) biological replicates ± SEM from three replicate experiments. (**B**,**C**) YAMC-AhR-KOs were treated with varying concentrations of DEHP for 24 h. Amplification was measured using SYBR green reagents. Gene expression was normalized by 18S. Mean (n = 3) biological replicates ± SEM from three replicate experiments. For all experiments, data are expressed as fold change relative to vehicle (0.1% *v*/*v* DMSO). Bars without a common letter are significantly different; *p* < 0.05.

The aryl hydrocarbon receptor (AhR) is a nuclear receptor that binds a diverse array of xenobiotic, natural, and endogenous compounds, promoting enzymes which catalyze their eventual degradation [22]. Through this process, reactive metabolites and free radicals are generated and when occurring in excess, can result in oxidative damage of organelles and intracellular structures [23]. YAMCs have been shown to possess a high degree of AhR bioactivity similar to that of human-derived colonocyte cell lines (i.e., Caco-2s), making them an excellent model to study receptor-mediated effects of ligands [24,25]. As DEHP and several of its metabolites have been shown to promote AhR activity in vitro we hypothesized that the receptor may mediate the effects of DEHP on both ROS production and apoptosis [26,27,28]. Therefore, we exposed YAMCs with a CRISPR-deleted AhR (YAMC-AhR-KOs) to DEHP (1, 10, and 100 µM) for 12 h and measured cleaved caspase activity. In contrast to Wild Type YAMCs (WT-YAMCs), a 12 h exposure DEHP at a concentration of 100 µM resulted in no significant changes to caspase activity (*p* = 0.3028), suggesting that apoptosis following DEHP exposure is due, in part to the AhR receptor activity (Figure 6A). Unlike WT-YAMCs, DEHP exposure (100 µM) to YAMC-AhR-KOs did not significantly alter *BAX* transcription (*p* = 0.1432; Figure 6B). Like WT-YAMCs, DEHP exposure resulted in no significant changes to *BCL2* gene expression relative to vehicle control (Figure 6C).

### 2.7. The Aryl Hydrocarbon Receptor, in Part, Mediates the Response to DEHP-Mediated Oxidative Stress

**Figure 7:**
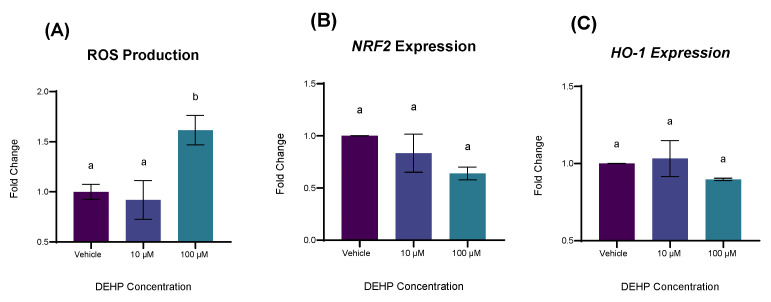
The effect of DEHP on ROS production and Oxidative Stress Genes in YAMC-AhR-KOs. (**A**) YAMC-AhR-KOs were exposed to varying concentrations of DEHP for 24 h. ROS activity was measured using the fluorescent probe DCFH-DA on a fluorescence plate reader (Agilent Biotek; Winooski, VT, USA). Mean (n = 9) biological replicates ± SEM from three replicate experiments. (**B**,**C**) YAMC-AhR-KOs were treated with varying concentrations of DEHP for 24 h. Amplification was measured using SYBR green reagents. Gene expression was normalized by 18S. Mean (n = 3) biological replicates ± SEM from three replicate experiments. For all experiments, data are expressed as fold change relative to vehicle (0.1% *v*/*v* DMSO). Bars without a common letter are significantly different; *p* < 0.05.

As with apoptosis, we further sought to measure ROS production and ROS-responsive genes in YAMC-AhR-KOs following DEHP exposure to characterize the role of the receptor in DEHP-induced oxidative stress. A 24 h exposure to DEHP at a concentration of 100 µM significantly increased ROS production in YAMC-AhR-KOs like WT-YAMCs (*p* = 0.0160; Figure 7A), yet the magnitude of difference was greatly subdued (approximately 3-fold vs. 1.5-fold difference in normalized fluorescence relative to vehicle control). In contrast to WT-YAMCs, DEHP exposure elicited no significant increase in the expression of either the *HO-1* or *NRF2* genes (Figure 7B,C), suggesting that DEHP-induced oxidative stress partly involves AhR activity.

## 3. Discussion

Despite regulation of its usage, DEHP is still used in a wide array of PVC-based plastic products that come into contact with humans, leading to continual exposure [2]. A broad collection of in vitro evidence highlights DEHPs toxicity in cell lines originating from reproductive, pancreatic, adipose, and hepatic tissue [29,30,31,32,33,34,35]. Yet, few studies to date have sought to investigate effects of DEHP exposure on cells originating in the large intestine, which is critical given that oral consumption is the primary means of exposure to the phthalate [1]. For the first time, we demonstrated that DEHP adversely affects cell growth patterns in non-malignant colonocytes, effects explained, in part, by reactive oxygen species-based intracellular signaling pathways. We further demonstrated that these effects may, in part, be the result of AhR-specific ROS production and pro-apoptotic signaling.

Apoptosis is a highly conserved and tightly regulated form of cell death, occurring in response to external or internal stimuli which damage organelles, DNA, or initiate specific apoptotic signaling pathways [36]. Using an assay that detects the activity of cleaved caspases 3/7 as an indirect measure of apoptosis, we demonstrated that short-term (12 h) DEHP exposure increased apoptosis in non-malignant colonocytes. We confirmed this finding at the transcriptional level by demonstrating that DEHP exposure increased the expression of pro-apoptotic gene *BAX* in conjunction with apoptosis induction, with no change in the expression of anti-apoptotic gene *BCL2*. This is consistent with data observed in cells derived from reproductive, pancreatic, muscular, and brain tissues [33,37,38,39,40]. DEHP exposure to denuded oocytes induced apoptosis as early as 1 h, demonstrated by Annexin-V/Propidium-Iodide quantified DNA breakage, increased protein expression of Bax, Bcl-2, and Cytochrome-C, and increased mRNA expression of *BAX*, *BCL2*, and cytochrome c (*CYCS*) [37]. In cultured rat granulosa cells, 72 h exposure to DEHP reduced cell viability, decreased mitochondrial membrane potential, increased the protein/mRNA expression of pro-apoptotic *BAX*, caspase 3 (*CASP3*) and *CYCS*, and decreased the expression of *BCL2* [38]. To date, only two in vitro studies have measured apoptosis in cell lines with colonic origins. In agreement with our findings, HCT-116 cells exposed to DEHP at concentrations of 32.5 μM, 65 μM, and 130 μM for 24 h decreased cell viability while concentrations of both 65 μM and 130 μM increased apoptosis [15]. In SW480 cells exposed to DEHP at a 10 μM concentration, no change in apoptosis was observed at either 24, 48, or 72 h [16]. Though conflicting, the concentration used in the second study (10 μM) may account for these differences [16]. Further, both the HCT-116 and SW480 cell lines originate from human colorectal carcinomas each harboring distinct mutation patterns that could contribute to differences in apoptotic behavior [17]. Thus, the conflicting evidence observed following DEHP exposure to SW480 cells may be a product of oncogenic signaling pathways affecting cell growth patterns in lieu of the phthalate exposure [17].

We demonstrated that short-term DEHP exposure to non-malignant colonocytes decreased the expression of *PIK3CA* and *AKT1*, genes involved in the PI3K/Akt/mTOR signaling cascade which upon activation promotes cell growth, metabolism, survival, and progression of the cell cycle [41]. PI3K and Akt negatively regulate Bax, preventing its translocation from the cytoplasm to the outer mitochondrial membrane, inhibiting apoptosis [42]. As we demonstrated that DEHP exposure induced caspase activity while enhancing the expression of the *BAX* gene, our findings suggest that downregulation of this pathway may contribute to DEHP-mediated apoptosis. The mechanism suggested here agrees with several in vitro studies elucidated in non-colonic culture systems. In mouse-derived muscle myoblast (C2C12) cells, DEHP exposure reduced the mRNA and protein expression of PI3K, AKT, and mTOR, simultaneously increasing both the mRNA and protein expression of Caspase-3 and Bax, while decreasing the mRNA and protein expression of Bcl-2 [43]. In mouse neuroblastoma-derived NS20Y cells, DEHP exposure reduced the mRNA and protein expression of PI3K and AKT, concomitantly increasing the mRNA and protein expression of Cyt-c, Bax, Bak, and Caspase-3, while decreasing the mRNA and protein expression of Bcl-2 [44].

In contrast to the apoptotic effects of DEHP in WT-YAMCs, we demonstrated that exposure to the phthalate in YAMCs with CRISPR-deleted AhR (YAMC-AhR-KOs) led to no such effects, suggesting the receptor plays a role in the pro-apoptotic effects of the phthalate. Certain AhR ligands have been shown to promote apoptosis through mechanisms principally involving receptor-mediated ROS production, yet studies investigating the specific role of DEHP in such a phenomenon are scarce [23,45]. DEHP exposure in vitro to mouse-derived primary neocortical neurons led to rapid ROS generation and transcription of AhR-target gene cytochrome P450, family 1, subfamily a, polypeptide 1 (*CYP1A1*); while the authors did not measure apoptosis in this study, they did demonstrate that DEHP reduced cell viability after prolonged exposure pointing to the possibility of a relationship between AhR activation, ROS production, and latent neuronal cell toxicity [27]. A similar finding was seen in Leydig cells exposed to DEHP metabolite MEHP; in this study, authors found that exposure to high-dose MEHP (100 μM) induced transcription of the *CYP1A1* gene, increased ROS production, and decreased cell viability after just 24 h of exposure [46]. Additionally, in mouse-derived primary antral follicle cultures intermediate dose MEHP (4 μM) disrupted follicular growth and induced the expression of *CYP1A1*, cytochrome P450, family 1, subfamily b, polypeptide 1 (*CYP1B1*), and aryl-hydrocarbon receptor repressor (*AHRR*); these effects were reversed with coadministration of AhR antagonist CH223191, linking antral follicular growth, AhR signaling, and phthalate metabolite exposure [47]. While we did not measure the expression of such genes in the findings presented here, we have shown YAMCs to possess a high degree of AhR bioactivity [24,25]. Therefore, we hypothesize that the effects of DEHP exposure in YAMC-AhR-KOs reported here are indeed reflective of a toxicant–receptor relationship. While beyond the scope of this manuscript, future findings are warranted to more comprehensively define AhR signaling dynamics during xenobiotic mediated apoptosis.

Several in vitro models of DEHP exposure have demonstrated that the phthalate increases the activity of p38 mitogen activated protein kinases (p38 MAPKs), stress-responsive MAPKs, which may inhibit cell cycle progression and promote apoptosis in a context-specific manner [48,49]. Importantly, activation of p38 MAPK-mediated apoptosis and cell-cycle arrest can occur downstream of AhR activation [45,50]. In H9C2 cardiomyocytes, exposure to AhR ligand kynurenine induced apoptosis and activation of MAPKs p38, c-Jun N-terminal kinase (JNK), and Extracellular signal-regulated kinase (ERK); such effects were attenuated following administration of AhR inhibitor CH-22319144 [45]. In agreement with this finding, in vitro cigarette smoke condensate exposure to GC-2spds(ts) spermatocytes inhibited DNA synthesis and cellular proliferation, which was accompanied by AhR activation and p38 MAPK pathway activation; pretreatment of cells with CH223191 attenuated p38 MAPK signaling and cell cycle arrest [50]. Taking these findings into consideration, the attenuation of caspase activity in our model of exposure may be, in part, a product of impaired MAPK signaling in the absence of AhR. This notion is further supported by our findings that DEHP decreased the expression of PI3K/AKT/mTOR pathway-associated genes (*PIK3CA* and *AKT1*), the activity of which has been shown to be negatively regulated by p38 MAPKs [51,52]. Therefore, crosstalk between the AhR receptor and stress-responsive signal transduction pathways can promote apoptosis in a ligand-specific and cell-specific manner and therefore may explain the AhR-dependent caspase activity observed in our study as well as those mentioned previously.

Reactive Oxygen Species (ROS) are molecules that are produced as byproducts of normal cellular metabolism [53]. In homeostatic conditions, tissues have competent defense mechanisms to eradicate ROS upon their production [54]. When ROS are generated to an extent whereby cellular defense mechanisms are incapable of reducing or quenching them, cells undergo a state of oxidative stress, leading to protein and organelle damage, and eventual cell death [53,54]. The transcription factor Nrf2 is a crucial initiator of cellular responses to oxidative stress or otherwise perturbed redox balance [55]. Under homeostatic conditions, Nrf2 remains sequestered to the protein Keap1 in the cytosol, which facilities its proteasomal degradation. Under conditions of oxidative stress, Nrf2 detaches from Keap1 and translocates to the nucleus, where it binds to genes with AREs in their promoter regions, such as those which code for antioxidant and ROS-scavenging proteins [55]. We demonstrated that DEHP exposure to non-malignant colonic epithelia increased the expression of Nrf2 target genes (*NRF2, GCLC, HO-1*), suggesting that DEHP exposure to non-malignant colonocytes increases intracellular oxidative stress, initiating a Nrf2-dependent response to restore intracellular redox balance. Indeed, we further demonstrated that DEHP exposure to nonmalignant colonocytes resulted in cellular ROS generation, suggesting that the upregulation of genes coding for antioxidant response proteins was likely a result of ROS-mediated Nrf2 activation and translocation to the nucleus. We confirmed the involvement of ROS production in DEHP-mediated apoptosis by demonstrating that pretreatment of non-malignant colonocytes with antioxidant NAC ameliorated apoptosis induced by DEHP. Our findings are in broad agreement with studies demonstrating that DEHP exposure increases ROS production in tandem with changes to Nrf2 transcription and/or expression. DEHP exposure to human neuroblastoma (SH-SY5Y) cells increased ROS production, Nrf2 expression, HO-1 expression and apoptosis, demonstrating that despite upregulation of the Nrf2 pathway by ROS such a response was insufficient to combat ROS production [56]. Therefore, our findings suggest that canonical antioxidant systems meant to quench ROS and inhibit oxidative stress were insufficient to counteract the insult of DEHP to the intracellular environment. This is further reinforced by our finding that DEHP induced the expression of the gene *CHOP*, a pro-apoptotic gene expressed during prolonged ER-stress and indicative of oxidative stress-induced organelle damage [57].

In contrast to these findings, we showed that DEHP exposure to YAMC-AhR-KOs led to no significant changes in either *HO-1* or *NRF2* transcription. We further demonstrated that despite DEHP significantly increasing ROS production in YAMC-AhR-KOs relative vehicle control, the magnitude of this effect was comparatively less than that of the equivalent exposure in WT-YAMCs. As mentioned previously, ligand activation of AhR and induction of CYP monooxygenases and other enzymes can contribute to oxidative stress; this, in turn, drives Nrf2-mediated promotion of antioxidant enzymes [22,58]. In our model, the lack of functional AhR and presumable decrease in activity of these enzymes could contribute to lower ROS production and by extension, decreased transcription of Nrf2-target genes. Alternatively, the promoter region of the *NRF2* gene contains xenobiotic response elements (XREs) inducible by AhR ligands [59]. Therefore, the decreased transcription of *NRF2* and *HO-1* in the absence of AhR may be emblematic of aberrant receptor signaling following DEHP exposure. Finally, as mentioned previously, AhR receptor can promote activity of the p38 MAPK signaling pathway, which has been implicated in apoptosis in models of DEHP exposure [60,61]. Interestingly, Nrf2 activation and translocation to the nucleus was shown to be dependent on AMPK/p38 signaling in mouse-derived BV-2 microglia cells exposed to toxicant sodium fluoride [62]. As MAPK pathways are sensitive to ROS production at large, the interrelationship between AhR, oxidative stress, and signal transduction pathways following DEHP exposure warrants further investigation [49].

We acknowledge several key limitations of our findings that provide the basis for future areas of investigation. We demonstrated that DEHP exposure induced functional and transcriptional measures of apoptosis in non-malignant colonocytes and linked these effects to ROS production and oxidative stress. Yet, the non-specific nature of the measures used in our model (i.e., measurements of non-specific ROS production, caspase activity) limit our understanding of the mechanism through which this occurred. Future studies should (a) consider the ROS subtype and source after xenobiotic exposure, (b) characterize additional functional measures of intracellular oxidative stress (i.e., lipid peroxidation, antioxidant enzyme activity), and (c) measure the activity of specific signaling pathways through which oxidative stress has been shown to drive apoptotic signaling. Additionally, our findings suggested that AhR activity played a role in the apoptotic and oxidative stress-inducing effects of DEHP in non-malignant colonocytes. Yet, our interpretation of these findings is limited, as we did not characterize the dynamics of the receptor in response to DEHP, nor did we characterize mechanisms in WT-YAMCs through which the receptor may have contributed to either apoptosis or oxidative stress in our model of exposure. Further studies should clarify this relationship by measuring the temporal dynamics of AhR activity following DEHP exposure and consider pathways through which the receptor may act to influence either apoptosis or oxidative stress. Lastly, most of our measurements were carried out over a short period of exposure, owing to our desire to capture transcriptional changes that may otherwise be difficult to capture with extensive exposure periods. To extend our methodology to a scenario with more relevance to chronic, human exposure, future studies should consider studying the intestinal effects of DEHP exposure over a longer duration of time, building off of the findings presented in this manuscript.

## 4. Materials and Methods

### 4.1. Cell Culture

Young Adult Mouse Colonocytes (YAMCs) and YAMC-AhR-KO cells were graciously provided by Dr. Robert Chapkin (Department of Nutrition and Food Science, Texas A&M University, College Station, TX, USA). These morphologically epithelial cells are non-malignant with no evidence of differentiation during culture [24,63,64]. YAMCs were cultured in RPMI 1640 (Corning; Corning, NY, USA) with 5% fetal bovine serum (FBS; HyClone; Logan, UT, USA), 0.1% Insulin, transferrin, and selenium (ITS; Sigma; Burlington, MA, USA); 1% penicillin/streptomycin; and 1% Glutamax (Gibco; Grand Island, NY, USA). Cells were maintained at 33 °C at permissive conditions, with 10 units IFN*γ*/mL (Sigma; Burlington, MA, USA) medium.

### 4.2. Chemicals and Dose Selection

≥99.5% Di(2-ethylhexyl) Phthalate (DEHP) was purchased from Sigma (D201154; Burlington, MA, USA). We chose a range of doses to capitulate the low and high ends of environmentally relevant (1–100 µM) and pharmacological (1 mM) exposures, such as those reported through food exposure or through exposure to medical devices and procedures like Extracorporeal Membrane Oxygenation (ECMO), hemodialysis, or blood transfusion [65,66,67]. DEHP was dissolved in dimethyl sulfoxide (DMSO; Sigma; Burlington, MA, USA), the final volume of which never surpassed 0.1% *V*/*V* in culture medium.

### 4.3. Cell Number

YAMCs were transferred to a medium containing 5% charcoal-dextran-stripped FBS, seeded at a density of 24,000 cells per well in 12-well plates, and grown at 33 °C. After 24 h, cells were exposed to DEHP at increasing concentrations (1 µM, 10 µM, 100 µM, 1 mM) or vehicle (0.1% *V*/*V* DMSO). Treatment-containing medium was replaced at 48 h. After 72 h of total exposure, a microscopy-based imaging method was used to count total cells across 25 representative areas of each well using a Cytation 5 Imaging Machine (Agilent Biotek; Winooski, VT, USA).

### 4.4. Proliferation

A Bromodeoxyuridine (BrdU) kit was used to quantify cellular proliferation (Millipore-Sigma; Burlington, MA, USA). YAMCs were transferred to a medium containing 5% charcoal-dextran-stripped FBS, seeded at a density of 2000 cells per well in 96-well plates, and grown at 33 °C. After 24 h, cells were exposed to DEHP at increasing concentrations (1 µM, 10 µM, 100 µM, 1 mM) or vehicle (0.1% *V*/*V* DMSO). Treatment-containing medium was replaced at 48 h. Then, 18 h prior to the conclusion of the assay, all cells were incubated with BrdU. After 72 h, BrdU incorporation into replicating cells was colorimetrically quantified with a spectrophotometer (Agilent Biotek; Winooski, VT, USA) and used as a measurement of cellular proliferation.

### 4.5. Cytotoxicity

Cytotoxicity was quantified with a colorimetric Lactate Dehydrogenase (LDH) assay (Roche; Basel, Switzerland). YAMCs were transferred to a medium containing 5% charcoal-dextran-stripped FBS, seeded at a density of 8000 cells per well in 96-well plates, and grown at 33 °C. After 24 h, cells were exposed to DEHP at increasing concentrations (1 µM, 10 µM, 100 µM, 1 mM), vehicle, or positive control (0.1% *v*/*v* Triton-X) for 24 h. LDH in the culture medium was used as an indicator of cytotoxicity and was colorimetrically quantified with a spectrophotometer (Agilent Biotek; Winooski, VT, USA).

### 4.6. Apoptosis

Cleaved caspase activity was used as an indicator of cellular apoptosis and was measured with the CaspaseGlo 3/7 Luminescence Assay (Promega; Madison, WI, USA). YAMCs were transferred to a medium containing 5% charcoal-dextran-stripped FBS, seeded at a density of 2000 cells per well in 96-well plates, and grown at 33 °C. After 24 h, cells were exposed to DEHP at increasing concentrations (1 µM, 10 µM, 100 µM), vehicle, or positive control (1 µM Staurosporine) for 12 h. Luminescence was measured at 12 h with a luminometer (Agilent Biotek; Winooski, VT, USA).

### 4.7. RNA Isolation

YAMCs were seeded in 100 mm dishes at a density of 30,000 cells per mL medium and exposed to concentrations of 10 µM, 100 µM DEHP, or Vehicle for 24 h. Trizol Reagent (Ambion; Waltham, MA, USA) was used to isolate RNA from cells and the purity of the RNA was measured using a Nanodrop 2000 (Thermofisher; Waltham, MA, USA). Sufficiently pure, eluted RNA was stored at −80 °C until usage.

### 4.8. cDNA Synthesis, RT-PCR, and Primer Selection

cDNA was synthesized from RNA using the Transcriptor First Strand cDNA Synthesis Kit (Roche; Basel, Switzerland). Following cDNA synthesis, Quantitative reverse transcription polymerase chain reaction (RT-qPCR) was performed using a 7500 Fast Real-Time PCR System (Applied Biosystems; Waltham, MA, USA) with SYBR Green Reagents for Amplification (Applied Biosystems; Waltham, MA, USA). Primer sequences used are listed in Table 1.

### 4.9. ROS Quantification

Dichlorofluorescein Diacetate (DCFH-DA) was used to quantify reactive oxygen species (ROS) in YAMCs after short-term DEHP exposure. YAMCs were transferred to a medium containing 5% charcoal–dextran–stripped FBS, seeded at a density of 2500 cells per well in 96-well black-walled (clear bottom) tissue culture plates, and grown at 33 °C. After 24 h, cells were exposed to DEHP at increasing concentrations (1 µM, 10 µM, 100 µM), vehicle, or positive control (50 µM H_2_O_2_) for 24 h. Next, the medium was aspirated and serum-free medium containing 20 µM DCFH-DA was added to each well and allowed to incubate at 33 °C for 30 min in the dark. Following this, cells were washed twice with serum-free medium and fluorescence was measured using a fluorescence plate reader (Agilent Biotek; Winooski, VT, USA). Fluorescence intensity was normalized to the number of cells per well.

### 4.10. N-Acetyl L-Cysteine Assay

YAMCs were transferred to a medium containing 5% charcoal–dextran–stripped FBS, seeded at a density of 2000 cells per well in 96-well plates, and grown at 33 °C. After 22 h, cells were pretreated with 5 mM N-Acetyl L-Cysteine (NAC) for two hours followed by a 12 h exposure to 100 µM DEHP, vehicle, or positive control (1 µM Staurosporine). Cleaved caspase activity was used as an indicator of cellular apoptosis and was measured with the CaspaseGlo 3/7 Luminescence Assay (Promega; Madison, WI, USA). Luminescence was measured at 12 h with a luminometer (Agilent Biotek; Winooski, VT, USA).

### 4.11. Statistical Analysis

All statistical analyses and figures were generated in GraphPad Prism version 10.5.0 for Windows (Graph Pad Software, La Jolla, CA, USA, www.graphpad.com). Following tests for normality (Shapiro–Wilk test), Means were compared using parametric or nonparametric tests and Outliers based on ROUT (Q = 1.0%) were excluded. Parametric methods included ANOVA followed by Tukey’s multiple comparisons test for comparing three or more means. Non-parametric methods include the Kruskal–Wallis (KW) test followed by Dunn’s multiple comparisons test for three or more means. Results were considered statistically significant when the *p*-value was ≤0.05.

## 5. Conclusions

Collectively, our findings suggest that DEHP exposure to non-malignant colonic epithelia results in abnormal elevations in ROS, leading to oxidative stress-induced apoptosis. Further, our findings suggest that these effects may be due, in part, to DEHP-mediated AhR activity. Due to the implications of this receptor, oxidative stress and alterations to homeostatic cell growth/death in the etiology of intestinal disease, our findings lay the mechanistic groundwork for future investigations studying DEHP’s gastrointestinal toxicity in vitro and serve as a starting point for follow-up studies. Future research should (a) expand on our short-term model to evaluate the long-term effects of DEHP on in vitro AhR bioactivity, oxidative stress and apoptosis in nonmalignant colonic epithelia, (b) clarify the nature of the oxidative stress response and ROS-mediated apoptosis observed in our findings, and (c) evaluate the activity and expression of cytoplasmic proteins involved in intracellular redox balance and signaling pathways defined in our study.

## Figures and Tables

**Table 1:** List of primers used in RT-qPCR.

Gene Name	Forward Sequence	Reverse Sequence
*BAX*	AATATGGAGCTGCAGAGGATG	CCAGTTGAAGTTGCCATCAGC
*BCL2*	GCTGGGGATGACTTCTCTCG	CCACAATCCTCCCCCAGTTC
*AKT1*	CTCATTCCAGACCCACGACC	TAGGAGAACTTGATCAGGCGG
*PIK3CA*	CACGACCATCTTCGGGTGAA	TCACGGTTGCCTACTGGTTC
*NRF2*	CTTTAGTCAGCGACAGAAGGAC	AGGCATCTTGTTTGGGAATGTG
*HO-1*	ATGTTGACTGACCACGAC	GCCCCACTTTGTTAGGAAA
*GCLC*	GGCCACTATCTGCCCAATTG	CTCCCCAGCGACAATCAATG
*CHOP*	TCTGTCTCTCCGGAAGTGTA	CTGGTCTACCCTCAGTCCTC
*DRP1*	TGCAGGACGTCTTCAACACA	GACCACACCAGTTCCTCTGG
*OPA1*	ATTGTCGGAGCAGGAATCGG	AGGATTGGCAGACTTCACAGG

## Data Availability

The data presented in this study are openly available in FigShare at the following link: https://figshare.com/articles/dataset/_b_The_Cellular_Effects_of_Di_2-ethylhexyl_Phthalate_in_Non-malignant_Colonic_Epithelia_Involves_Oxidative_Stress_b_/29922599?file=57220043 (accessed on 29 November 2025).
